# The Physiology of Sweating

**Published:** 1880-01

**Authors:** 


THE PHYSIOLOGY OF SWEATING.—Sweating can be induced by reflex action, and also in a very marked and singular manner by jaborandi, and by the active principle of that drug—pilocarpin. In from three to five minutes after the subcutaneous injection of a solution of hydrochlorate of pilocarpin, in man, the flow of saliva increases, perspiration appears, first on the head, and then gradually over the whole body, and lasts about an hour, or, if the patient be in bed, for two or even three hours. This effect, Luchsinger thinks to be due to the pilocarpin acting as a direct stimulant to the nerve centres. He tied the abdominal aorta in a cat, and then injected pilocarpin into a vein. Under these conditions the pilocarpin was unable to reach the glands in the posterior extremities, and thus to act as a direct stimulant; nevertheless the feet were soon bathed in sweat. Atropin inhibits the secretion of sweat, for if after the injection of one one-hundredth of a gramme of pilocarpin, three one-hundredths of a gramme of atropin be injected, the commencing perspiration is arrested in about ten minutes. If now a hundredth of a grain of pilocarpin be injected into one of the feet, beads of sweat burst forth on this foot; but the rest of the body being still under the influence of atropin remains dry.

An acid reaction is generally attributed to this secretion, but Luchsinger and Truempy have ascertained that in man as well as in the cat the reaction is really alkaline, and that the acidity which has been observed is due to the fact that the secretion of the sebaceous glands is ordinarily acid, or rather becomes so in the action of decomposition to which it is prone.—*London Lancet.*

